# Advertisement

**Published:** 1848-01-01

**Authors:** 


### ADVERTISEMENT.

It is only recently that Mental Philosophy and Physiology have been practically studied. The subtleties and scholastic mysticism which formerly obscured our metaphysical researches, have been dissipated by a more enlightened method of induction. The discoveries which have been made in Physiology and Pathology have revealed to us new and important principles connected with the constitution of the human mind, both in health and in disease. Hence the most valuable practical results are being now daily developed, not only in private practice, but especially in those public Institutions which are dedicated to the treatment of Mental Disease. It has therefore been suggested, that a Journal, devoted to the progress of Psychological Science, will not fail to be acceptable to all who are interested in this department of knowledge. We do not wish to deal with the Science of Psychology—which embraces, in its widest sense, an investigation into the normal and abnormal conditions of the human mind—as mere theorists. The knowledge which does not admit of practical application to the comforts or the wants of suffering humanity may be curious, but is in a great measure valueless.

The aberration of the human intellect is that which, above all other calamities, has the strongest claim upon our sympathies; and they only who, devoted to this branch of professional study, are accustomed to watch the clouding and unclouding of the mind, under the shadow of disease, can discriminate the phases through which it passes, and the degree [illegible]estoration of which it may be susceptible. They only can from their experience acquire correct principles of diagnosis; and it is thought desirable that a Journal should be established specially devoted to their communications,—which should enable them to make known the results of their practice, and the suggestions and discoveries which may from time to time occur to them,—record judicial proceedings affecting the question of Insanity, and moral responsibility, occurring before our civil and criminal tribunals,—and open a field for fair and impartial discussions on all subjects relating to the Physiology and Pathology of the Human Mind. Such a Journal we hope to supply in the periodical now announced.

The Journal of Psychological Medicine will contain Analytical and Critical Reviews.

These Articles will aim at conveying a concise and impartial abstract of all new works, domestic and foreign, on Psychological subjects. To concentrate carefully the knowledge diffused over many pages, to select useful information, and expose unsound theoretical views, to deal justly and conscientiously with all publications that come under notice, will be the object of our ambition. We shall critically investigate, and fearlessly pronounce judgment, on matters which come under Review, but in so doing, we shall not affect that hypercriticism which rejoices in the brilliancy of the wit with which it assails and victimizes the object of its attack. The interests of Science require no such display of intellectual gladiatorship. We shall seek only to impart information; and in all cases to pronounce judgment on doubtful or disputed points, fairly and without acrimony; for a critical analysis on subjects connected with Mental Philosophy ought to be as carefully and rigidly conducted as any analytical experiment in Practical Science.

We have reason to know that among our professional brethren who are already interested in Psychological investigations, a mass of valuable information exists, which is individualized and scattered for the want of a Journal recognised as the organ of such communications. We therefore cheerfully invite their co-operation; and have great satisfaction in stating, that we have been already favoured with contributions from many of the most eminent members of the profession.

A careful abstract of the Reports of Public and Private Asylums in Great Britain, France, Germany, Prussia, and America, will be an important feature of this Journal. The simultaneous publication of these, in a condensed form, will constitute a valuable body of evidence on the present state of Lunacy, and will not fail to be suggestive of many practical improvements in the constitution and management of these Institutions.

It is only of late years that the voice of humanity has been permitted to plead before the tribunals of justice in behalf of the criminal lunatic. It is obvious that the reports of such trials, the evidence upon which cases for future precedents are established, ought to be recorded with great care. So, likewise, the Commissions of Lunacy, so frequently held to inquire into the mental competency of persons to administer their affairs, ought, in like manner, to be carefully, or rather officially reported. Cases also frequently occur demanding special notice, which animadvert on doubtful evidence, and convey information which may enlighten and guide juries in coming to a proper verdict. The most extraordinary ignorance prevails on the subject of Insanity, and it will be one of the objects of this department of the Journal to give an exposition of those Psychological principles in the consideration of Insanity which should guide jurors in the discharge of their solemn duties.

In Germany, France, and lately in America, Psychological researches have been pursued with more zeal than in this country; and articles which deserve more than an ephemeral existence constantly appear scattered through different periodicals. Translations and abstracts of these will appear in this department of the Journal, and we shall be guided, in making our selections, by the practical value of the articles, rather than by their speculative ingenuity.

Under the head of Correspondence, Domestic and Foreign, the Journal will be open to discussion on speculative as well as practical subjects. We do not invite controversy, but shall throw open our pages to fair, free, and independent inquiry, satisfied that Truth, to carry out its own divine mission, must breathe an atmosphere of freedom.

Edited upon these principles, we have reason to believe that the JOURNAL OF PSYCHOLOGICAL MEDICINE will supply a *desideratum* which at present exists in professional literature, and we are fully satisfied that the success of the undertaking will depend upon the ability with which the Editor will be enabled to realize the expectations which he has in the above remarks held out.

In endeavouring to establish a Journal devoted exclusively to the consideration of the Human Mind in its abnormal state, we feel that we are breaking new ground in this country, and that many difficulties will beset our path; but the anticipation of these obstacles does not in the slightest degree damp our energies, or lessen our hopes of a triumphant result.

N.B.—It is the intention of the proprietor of this Journal that it shall consist of nine sheets—144 pages. Owing to a pressure of important matter, the first number exceeds twelve sheets, which it is evident cannot be sold at the price announced without loss. The succeeding numbers will contain nine sheets, as originally proposed.

